# “You get a better idea of what you want to do with your life”: needs and experiences of transgender and gender diverse individuals participating in an internet delivered emotion regulation treatment

**DOI:** 10.3389/fpsyt.2026.1659076

**Published:** 2026-02-05

**Authors:** Markus Byström, Hedvig Engberg, Hanna Sahlin

**Affiliations:** 1 Centre for Psychiatry Research, Department of Clinical Neuroscience, Karolinska Institutet, Stockholm, Sweden; 2 ANOVA, Andrology, Sexual Medicine and Transgender Medicine, Karolinska Universitetssjukhuset, Stockholm, Sweden; 3 Department of Women’s and Children’s Health, Karolinska Institutet, Stockholm, Sweden; 4 Medical Unit Gynecology and Reproductive Medicine, Karolinska University Hospital, Stockholm, Sweden

**Keywords:** emotion regulation, gender diverse, internet-delivered treatment, reflexive thematic analysis, transgender

## Abstract

**Background:**

Transgender and gender diverse (TGD) individuals experience elevated rates of emotional distress, often thought to be linked to minority stress, identity-related challenges, and limited access to affirming mental health care. Emotion regulation has emerged as a potential key therapeutic target for improving well-being in this population but less is known about how TGD individuals describe their own needs in relation to psychological treatment.

**Aim:**

This study aimed to qualitatively explore the emotional and psychological needs of TGD individuals, and their experiences of an internet-delivered emotion regulation intervention (I-ER) specifically developed for TGD people.

**Method:**

Ten TGD individuals who had received the I-ER treatment participated in semi-structured interviews. Data were analyzed using reflexive thematic analysis.

**Results:**

Four themes were identified: (1) Emotional Exploration and Self-Understanding, (2) From Emotional Insight to Change, (3) Possibilities for and Limitations in Social Support, and (4) Barriers and Bridges to Treatment Engagement. Participants described the intervention as helpful for emotional insight, identity exploration, and behavioral change. The TGD-specific adaptation fostered a sense of recognition and belonging for most participants. However, challenges related to structure, support access, and individual fit were noted.

**Conclusion:**

Emotion regulation interventions tailored to TGD individuals can be perceived as helpful and supportive when culturally grounded, flexible, and relationally sensitive. Findings highlight the importance of integrating identity-affirming content and addressing diverse support needs in digital mental health programs.

## 1 Introduction

People whose gender identity or expression does not correspond to the sex assigned at birth are commonly referred to as transgender and gender diverse (TGD) (1). According to population-based studies, TGD people are at a higher risk for mental health problems such as depression and suicidality compared to cisgender (i.e., non-transgender) individuals (2). The discomfort, significant distress, or impairment caused by the incongruence between the sex assigned at birth and gender identity, commonly referred to as gender dysphoria (3), is thought to be a contributing factor for this increased risk. Other factors, such as social stress stemming from experiences of victimization, discrimination, social exclusion, invalidation of inner experiences, and other threats to social safety related to gender nonconformity, have also been proposed as contributing factors to TGD individuals’ increased risk of mental health problems (4, 5).

Even though the causes of TGD individuals’ heightened risk of mental health problems remain uncertain, readily available psychological therapies aimed at preventing mental problems among TGD individuals may lessen suffering in this vulnerable population. For this reason, psychological interventions should be taken into consideration in the provision of care for TGD people according to established standards of care (1). Such interventions might be provided during the process of transitioning, which includes social, medical and legal aspects through which TGD people may affirm their gender identity. Social transition typically refers to changes in gender expression and social recognition, such as adopting a new name or pronouns, altering appearance or presentation, and communicating gender identity to others. Medical transition refers to medical or surgical interventions intended to align physical characteristics with one’s gender identity. Legal transition encompasses the process of changing one’s name and gender marker on official documents. Not all TGD individuals engage with all these aspects of transitioning, rather individual pathways are shaped by personal needs, access to care, legal structures, and social context. However, due to the lack of research examining psychological treatments that explicitly target the needs of TGD persons, it is difficult to determine what kind of treatments should be provided (6). This may partially be explained by the fact that treatment targets for TGD individuals historically have been ill-defined (7), or even harmful when they have focused on changing TGD peoples’ gender identities, i.e. conversion therapy (8). Even well-intended treatment efforts have left many treatment seekers feeling rejected, misunderstood, or even discriminated against, due to a lack of understanding and insight into the lived experience of TGD people (9). Further, it is important to carefully consider what constitutes affirmative therapies (those that validate, support, and affirm a person’s gender identity and expression, rather than questioning or pathologizing it) for TGD individuals in order to avoid individualizing and pathologizing structural problems when applying psychological theories on lived experiences (10). In light of this, there is a need for comprehensive, knowledgeable, and supportive psychosocial treatments that could reduce mental health problems of TGD people.

Prior studies have shown that social support affects psychological distress in TGD people, being a risk factor when it is absent (2) and a protective factor when it is present (11). Without adequate social support in identity development and transition in their larger social context, TGD people may continue to experience distress (12). Further, TGD people experience general stressors alike their cisgender counterparts, however they are also exposed to specific stressors associated with belonging to a stigmatized minority (13). Such stressors could potentially strain the coping and adaptive emotion regulation abilities of TGD people (13, 14). Emotion regulation centers around the ability to manage one’s emotions, especially their intensity and duration, in order to attain important personal goals and meet external demands (15). Pervasive invalidation, i.e. having one’s internal experiences (e.g., emotions, cognitions, desires, and other private behaviors), external expressions (e.g., overt or public behaviors), or core sense of self (including self-initiated actions and identity) negated, dismissed, or devalued, can impact the ability to regulate emotions (16), and it has been suggested that even people with a low biological susceptibility to emotion dysregulation may develop difficulties regulating their emotions if they experience persistent invalidation (17). In this way, emotion dysregulation in TGD persons may be influenced by persistent invalidation within the dominant culture (12, 18). A recent study found that emotion regulation skills mediated the relationship between stigma-based victimization and suicidality in TGD people (19). This implies that improving emotion regulation abilities could be a key therapeutic target in psychological treatment for TGD people that may help mitigate the mental health disparities described in previous research. This therapeutic approach may be beneficial regardless of whether emotion regulation difficulties are caused by minority stress, or if minority stress in conjunction with general stressors increase the overall stressors for TGD people, or whether a third factor, such as genetics, is associated with being TGD and emotion regulation difficulties.

A few prior studies have evaluated psychological interventions aimed at enhancing the mental health of TGD people. These treatments adopt an affirmative, cognitive-behavioral approach showing trends of being effective in improving mental health (20, 21). To the best of our knowledge, psychological therapies that specifically target enhancing emotion regulation abilities have not been evaluated for TGD people. Previous research by Eyssel et al. (22) that used surveys in a non-clinical online sample explored how TGD people (n=415) described their own mental health needs. Nearly two-third of participants considered mental health counselling to be helpful during their transition, with a majority viewing integrated mental health counselling within specialized transgender health care centers to be positive and a large majority viewed the possibility for telemedical treatment as beneficial (22).

### 1.1 Objective and aim

Against this background, there is limited knowledge regarding TGD individuals’ specific needs and experiences in psychological treatment. Although previous research supports the use of telemedical psychological interventions in specialized transgender health care, further exploration of participants’ perspectives remains warranted. Qualitative studies add important perspectives in such exploration. Building on our prior feasibility evaluation of an internet-delivered emotion regulation treatment (I-ER), the aim of the current study was therefore to qualitatively explore TGD individuals’ needs and experiences of participating in the I-ER treatment.

## 2 Methods

The current study was a qualitative interview study exploring the needs and experiences of TGD individuals who had participated in the feasibility study of I-ER (23). The treatment was specifically developed for TGD individuals and included an optional parallel intervention offered to a close other (e.g., family member, partner) of the TGD individual. Those eligible for participation in the I-ER study were TGD individuals aged 18 or older, who were currently undergoing assessment of gender dysphoria at ANOVA (a center for sexual medicine, andrology, and trans medicine at Karolinska University Hospital, Stockholm, Sweden), as well as their close others. For more details, see Byström et al. (23). The inclusion criteria for the current study were having received the I-ER intervention and being willing to participate in a qualitative interview.

Reflexive thematic analysis (reflexive TA) as described by Braun and Clarke (24) was used within the study. This analytical method was chosen for its flexible and open analytical approach, which is suitable for exploratory research, where prior knowledge about the research question is limited. Within reflexive TA, the researcher’s active role in development, analysis, and reporting of patterns in the data is acknowledged, and as such there is a need for transparency about the researchers’ theoretical assumptions (24). In this study, interviews were coded inductively from an experiential position, where participants’ experiences and needs were analyzed at a semantic level. Further, all authors are health care practitioners with experience of working with TGD individuals, MB and HS are clinical psychologists, and HE is a medical doctor specialized in gynecology. These clinical roles and expertise in different academic fields have informed the planning and execution of the present study. The study was conducted at ANOVA, a clinical unit assessing and treating gender dysphoria where MB also works clinically with evaluation and treatment.

### 2.1 Procedure and participants

All participants from our I-ER feasibility study (23) were invited to participate in a qualitative semi-structured interview and 10 participants accepted. Interviews were conducted between post-treatment and the 3-month follow-up assessment. During the time of the interviews all participants were still undergoing an assessment for gender dysphoria at ANOVA. According to national guidelines in Sweden (25) these assessments are individualized and conducted by a multiprofessional team. The assessment varies in length and number of visits but usually includes at least eight visits. At the time this study was conducted, the assessment process spanned over a period of at least one year, partly due to long wait times (26). Some participants had attended a single visit and were waiting to see a psychologist, while others had attended multiple visits and had an ongoing contact with a psychologist conducting their assessment. Demographic information as well as participants’ experiences of gender-affirming treatments and social transition, was collected thorough self-report forms. Out of the 10 TGD individuals who were included in the study, 60% were assigned male at birth and 40% were assigned female at birth. In terms of gender identity, 50% of the sample consisted of trans women, 30% of trans men and 20% of non-binary individuals. Participants had a mean age of 30.5 years (SD = 9.17), with ages ranging from 19 to 46 years. Half of the participants reported being partnered, and a large majority indicated that they grew up in Sweden. Educational attainment varied in the study sample where a majority of the participants had some form of post-secondary education. Regarding occupational status, about half of the participants worked full time and about half were engaged in studies, while some were unemployed or working part-time. Almost all participants had undergone some degree of social transition, and a majority reported having fully socially transitioned, with about a third reporting that they had partially transitioned socially. Among those who had fully socially transitioned, the average duration since they started their social transition was 1.25 years (SD = 0.92). 20% of participants had initiated hormone replacement therapy (HRT), and for those individuals, the average duration on HRT was 3 years. Participants had completed the internet-administrated emotion regulation treatment to varying degrees, but on average participants had completed 7.3 modules (SD = 2.87) with a range of 2–10 modules completed. Six out of the 10 participants in the current study had a close other in the optional, parallel intervention during treatment. Close others included parents, siblings, partners, and friends.

### 2.2 Data collection

Individual semi-structured interviews were performed in person or via secure video call. All interviews were conducted between March and August 2023 in Stockholm, Sweden. The interviews were audio recorded and averaged at 39 minutes. The interview guide was developed by all authors. MB conducted 8 of the interviews, while HS conducted the remaining 2. Before the interviews, participants were given time to review the consent form and provide their informed consent. Each interview started with shorter background questions and progressed to open-ended questions about experiences of participating in the I-ER and needs before and after treatment with suggestions for follow-up questions in the interview guide. To maintain an awareness of subjective bias and emotional responses on the interview situation and analysis, memos were made continuously to document the recurrent discussions throughout the interview and analysis phase of the research.

### 2.3 Intervention

The internet-delivered emotion regulation treatment, I-ER which was developed and evaluated in a feasibility study (23), was based on the minority stress model (27) adapted for TGD individuals (13) and emotion regulation as defined by Gratz and Roemer (15). The treatment had three distinct sections: an introductory part covering the minority stress model followed by content on identity and identity development before several models covering emotion regulation. For a more detailed description of treatment content, see **Table 1**. Before inclusion, participants were informed that the aim of the treatment was not to influence their gender identities or attitudes toward gender-affirming medical treatments, but rather to strengthen emotion regulation abilities. During treatment development, non-governmental organization members advocating for TGD individuals were consulted and provided feedback on content to ensure that the treatment’s focus and goals aligned with the needs of the target population.

**Table 1:** Treatment content for TGD individuals.

Module	Content
1. Introduction	Introduction to the treatment, psychoeducation on gender minority stress model and emotion regulation. Work sheet on minority stress and emotion regulation skills.
2. Identity	Psychoeducation about identity and identity development and its’ stages, gender identity as well as validation/invalidation. Work sheet on identity development and situational analysis.
3. Emotional awareness	Psychoeducation on emotion regulation strategies and emotions. Work sheet on emotional awareness and situational analysis.
4. Functions of Emotions	Psychoeducation on emotions, primary and secondary emotions as well as clear and cloudy emotions. Work sheet on primary and secondary emotions and situational analysis.
5. Emotion regulation abilities	Psychoeducation on emotional regulation strategies in relation to secondary and cloudy emotions as well as tolerance of uncertainty. Work sheet on emotion regulation strategies and situational analysis.
6. Relational skills	Psychoeducation on relational skills. Work sheet on relational skills and situational analysis.
7. Values	Psychoeducation on values. Work sheet on values.
8. Valued direction and commitment	Psychoeducation on valued direction, commitment and goals tied to values. Work sheet on valued actions and situational analysis.
9. Emotional regulation abilities, relational skills, and valued direction	Repetition of themes in previous modules. Works sheet on combining different skills and strategies and situational analysis.
10. Relapse prevention plan	Psychoeducation on relapse prevention. Work sheet on establishing a relapse prevention plan.

The treatment was asynchronously delivered via a secure web-based treatment platform (BASS). The treatment consisted of 10 consecutive modules and participants were encouraged to work with one module each week during a period of maximum 12 weeks. Each participant was assigned an individual therapist (licensed psychologist or psychology student under supervision) who provided weekly written feedback on assignments and were available via messages on the platform. When participants had handed in assignments within a module, their assigned psychologist either encouraged them to go back and continue working with the same content or provided access to the next treatment module. Each module consisted of written material to read with corresponding worksheets and homework assignments. The length of modules varied between two to six and a half pages (A4) with an average of four pages. Close others had an individual log-in to the same platform and could access six modules during the same time-period as the TGD individuals participating in treatment. The intervention for close others was administrated in the same manner as it was for TGD individuals, and close others likewise had an assigned individual therapist. Content included psychoeducation on the gender minority stress theory, emotion regulation strategies, validation and interpersonal skills, see **Table 2** for a more detailed description. The length of modules in the close other intervention varied between three and a half to eight pages (A4) with an average of five pages.

**Table 2:** Treatment content for close others.

Module	Content
1. Introduction	Introduction to the treatment, psychoeducation on gender diversity and gender dysphoria, the minority stress model and emotion regulation. Work sheet on emotional reactions, distress and worries.
2. Emotion regulation	Psychoeducation on emotion regulation strategies and emotions. Work sheet on emotional awareness and information from emotions.
3. Primary and secondary emotions	Psychoeducation on emotions, primary and secondary emotions as well as clear and cloudy emotions. Work sheet on primary and secondary emotions and situational analysis.
4. Validation	Psychoeducation on validation, invalidation and emotional regulation difficulties, self-validation, relational validation. Work sheet on self-validation and relational validation.
5. Relational skills	Psychoeducation on relationships and emotion regulation, maladaptive relational patterns, adaptive relational skills. Work sheet on emotion regulation in a relational context and validation.
6. Summary	Reiteration of emotion regulation, validation, adaptive relational skills, values, forging a path forward and focus on continued process. Work sheet summarizing treatment content and planning for the future.

### 2.4 Analysis

Transcription of the interviews was assisted by Whisper (28), resulting in drafts of orthogonal transcriptions. These drafts were then reviewed by MB who listened through the interviews and made corrections in the drafted transcriptions to ensure accuracy in accordance with guidelines by Braun and Clarke (29). A reflexive thematic analysis (rTA) was conducted following the six-step approach described by Braun and Clarke (24) in Nvivo version 14.24.2. These six phases are: 1. Familiarizing yourself with your data; 2. Generating initial codes; 3. Searching for themes; 4. Reviewing themes; 5. Defining and naming themes, and 6. Producing the report. After reading the entire data set, thoughts about initial codes and possible patterns were discussed with the two other authors before the coding began. Initially, codes were intended to summarize the statements made by respondents into descriptive categories. In the next step, these initial codes were compared and revised, as well as grouped into clusters. During revision of initial codes, some codes were found to have identical meanings but had slightly differing names and some codes had different names but were found to identify similar concepts. Such codes were combined and renamed. During the comparison and revision process, additional codes were also created. All revised and new codes were then applied to the entire data set again. During the grouping process, the authors discussed potential relationships between clusters and how they could be combined into themes. Candidate themes were conceptualized and reviewed, compared and tested against each other to check for internal homogeneity and external heterogeneity (30). After this, themes were refined, sub-themes were defined, and names were given to each theme and sub-themes before the last step where the analysis was written up in the report.

### 2.5 Quality standards and trustworthiness

In order to ensure quality and trustworthiness of the analysis, the study used the 15-point checklist of criteria for good thematic analysis (30). Other quality-control methods such as using independent coders, member checking, and triangulation was not employed in the study as these methods mainly focus on analytical consensus, which does not align with reflexive TA. Instead, reflexive TA emphasizes the researcher’s active role in the knowledge production and reporting of rich and complex results that capture more than one analytic observation (31). Trustworthiness of the results in the current study therefore rely more heavily on the authors differing expertise from varied academic and clinical disciplines and our collective clinical experience with the patient group that we used to gain a deeper understanding of the material.

### 2.6 Ethical considerations

During recruitment, potential participants were informed about the aim of the study, its affiliation with Karolinska Institutet, that participation would be voluntary and confidential, and that personal details would be excluded from the finalized manuscript. Prior to the commencement of the interviews, participants were informed about the use of data. All participants gave their written informed consent. The study has received ethical approval by the Swedish Ethical Review Authority, Dnr: 2022-00577-01, with amendments Dnr 2023-00601–02 and Dnr 2023-04964-02.

In relation to the historical context where psychotherapy for TGD individuals has been used in attempts to convert gender expression or identities (7, 8), it was important to explore how participants perceived the treatment content. Ethical research also demands engagement with the communities involved, and for this reason patient organizations were involved in the treatment development stage of the I-ER feasibility study (23), following best practices outlined by the European Professional Association for Transgender Health (32).

## 3 Results

By analyzing the ten interviews, four themes, and two sub-themes related to needs and experiences of TGD individuals were developed. Theme four: Barriers and Bridges to Treatment Engagement surfaced repeatedly across the dataset, not as an overarching theme, but as cross-theme insights. An overview of themes and sub-themes with illustrative quotes is provided in **Table 3**.

**Table 3:** Overview of themes with illustrative quotes.

Theme	Sub-theme	Illustrative quotes
1. Emotional Exploration and Self-Understanding	*1.1 Searching for Understanding*	R6: “I was very confused about myself. I have a very hard time putting feelings into words and explaining them. I hoped the treatment would help me with that.”
	*1.2 Seeing Myself More Clearly: Emotional and Identity Insight*	R2: “Trying to understand how you have formed your identity and how you relate to society sometimes in relation to your identity and the importance of belonging to deal with minority stress to a certain degree, I think I definitely strengthened some skills.”
2. From Emotional Insight to Change		R4: “I’ve started to dress a little bit more like I do at home outside, so I somehow make myself visible to others. I think I’ve tried to hide, or I think, I know, that in the past I’ve hidden from my workplace and friends and family. I have a self-perception of who I am, and I have always thought that everyone else can see that just as well as I can see it. I also thought that no one liked me for that. So, I hid in some ways, maybe dressed differently or lied about who I am to others. It’s been tough to have to do that, but now it’s completely different. I dress how I want to and I’m not afraid to express myself anymore.”
3. Possibilities for and Limitations in Social Support		R5: “I invited my mom, and she has always been very supportive of me, but I think she was very happy with it anyway, that she was able to get a better understanding of her emotions and understand things better I think.”
4. Barriers and Bridges to Treatment Engagement		R2: “It was very rewarding that there were many concrete examples that felt very correct and relevant for trans people. It felt very based in something real, that it was not taken out of thin air, but things I recognize, things I have talked to other trans people about. I felt more seen in knowing that there is understanding in psychiatry for these things, it felt very hopeful and positive.”
		R1: “I found the material I had to work with very optimistic in relation to what it’s like to be a trans person in today’s political and social climate”.

### 3.1 Theme 1: emotional exploration and self-understanding

This theme contains two sub-themes. The first subtheme: Searching for Understanding encompasses respondents’ descriptions of needs they had when they entered the treatment. Sub-theme 2: Seeing Myself More Clearly: Emotional and Identity Insight contains respondents’ reflections on how these needs were met by the treatment.

#### 3.1.1 Sub-theme 1: searching for understanding

In reflecting on what had led them to participate in the I-ER treatment, respondents described varying needs, oftentimes in more general terms, such as wanting to understand themselves or their emotions better.

R6: “I was very confused about myself. I have a very hard time putting feelings into words and explaining them. I hoped the treatment would help me with that.”

For many, these needs were not framed in clinical terms, for example by describing themselves as depressed or having anxiety, but rather as difficulties in processing, understanding, or expressing emotions. This initial motivation often reflected broader experiences of emotional overwhelm or dysregulation.

R10: “I am going through a very difficult period right now during the winter with severe emotional overload.”

R4: “I have had a lot of difficulty dealing with emotions but also dealing with situations that trigger emotions and that lead to certain behaviors.”

Some respondents described more specific reasons for participation that related more closely to clinical distress, such as experiencing social anxiety and stress, but did not necessarily frame this in relation to specific psychiatric diagnoses. One respondent reflected on how they thought these specific problems were related to minority stress.

R2: “I have some problems with social anxiety and stress and social phobia and stuff like that, so it felt very relevant to that, I think. Just dealing with stress, maybe specifically minority stress.”

Beyond minority stress contributing to experienced problems, respondents also related needs prior to starting treatment to experiences of gender dysphoria, specifically in social situations that they hoped could be alleviated by receiving treatment.

R5: “I experience gender dysphoria and quite a lot of social dysphoria and I go to a school right now where I don’t really feel like myself … or where it doesn’t feel natural to be out with my identity and what I was looking for was strategies or ways to facilitate that.”

As both minority stress and gender dysphoria were part of many respondents’ initial motivation for participating in treatment, needs were often described to specifically relate to respondents’ trans identities. Beyond individual needs, several respondents described a wish to contribute to and further research that could benefit TGD individuals, R1: “I also have an interest in helping science wherever possible”. As this was recurrently described, a broader wish to further science was clearly part of the initial motivation for many respondents. For some, this was described as an act of solidarity, R9: “I thought it was good for research, representation as well as an act of solidarity”.

Initial needs were in this way often rooted in an experienced emotional ambiguity and a desire for self-understanding, and oftentimes respondents expressed that by sharing these experiences they hoped to help others in similar situations. Treatment was seen as an opportunity to develop emotional literacy, particularly within the context of TGD identity and social stressors.

#### 3.1.2 Sub-theme 2: seeing myself more clearly: emotional and identity insight

In reflecting on the emotion focus of the treatment, respondents expressed that the treatment not only addressed emotional regulation and helped them understand their emotions better but felt that this also supported a deeper process of self-understanding. Several respondents described an increased ability to identify, name and express their emotions. This process of gaining a more nuanced emotional literacy affected respondents directly but it also affected how confident they felt in being able to convey their experiences to others.

Respondents described how the treatment’s explicit focus on minority stress helped them gain perspective on their own experiences, allowing them to articulate aspects of their lives they had previously not been able to describe in much detail.

R2: “The treatment was very good at educating on what minority stress means. In many ways it was very educational and informative to begin with that, so that you understand how that relates to what you may have only talked about as social stress before.”

By gaining language to articulate their experiences as well as a deeper understanding of how their contexts affected them, respondents described relating to themselves and their feelings differently. Further they reflected on how working with emotion regulation not only affected how they understood their own emotions, but also how they related to themselves with recounts of increased self-compassion and self-confidence. These experiences were described to enact more encompassing changes.

R5: “A little more, what can I say, compassion for myself or understanding that I get tired and stressed quite easily.”

Many respondents described how working with identity development, where they were encouraged to describe and reflect on what had helped shape them into who they are, gave them new perspectives on processes that they previously related to with less awareness.

R6: “It helped me a lot to put into words, among other things, this assumed identity. I’ve found that I’ve used it a lot over the years, so it was really an eye-opener.”

This process of reflecting on identity development was described by many respondents to feel both affirming and personally meaningful, helping them develop skills they needed. In being able to reflect on how they had formed their identities, respondents also reflected on how this related to their social contexts and experienced problems stemming from minority stress.

R2: “Trying to understand how you have formed your identity and how you relate to society sometimes in relation to your identity and the importance of belonging to deal with minority stress to a certain degree, I think I definitely strengthened some skills.”

In this way, being able to reflect broadly on identity development and feeling seen in treatment was important for many respondents as this created a safe space where they felt seen. This allowed them to take a reflective and explorative stance while working with the treatment content which contributed to their ability to gain new perspectives.

### 3.2 Theme 2: from emotional insight to change

This theme contains participants’ narratives of observable behavioral changes that emerged as they developed greater emotional awareness and a more nuanced understanding of themselves. Recurrently, respondents described how gaining emotional insight facilitated real-world changes in their behavior and communication, allowing them to act in ways that were more congruent with their identities.

R8: “I have probably become braver, and I stand up for myself. Partly because of what I have read in the treatment.”

As respondents gained clarity about their emotions and felt more confident in being able to describe their experiences, many reported being able to communicate with others better.

R3: “I’ve definitely gotten better at communicating with my friends, talking to them about my day, for example. Things like that.

I: Has that affected your relationship with your friends in any way?

R3: Yes, I think so. It’s still a bit scary to do it, but it definitely feels like it has had a positive impact.”

In some cases, this extended into changes in visible expressions of gender identity, such as changes in clothing or behavior in social settings, actions that respondents connected to feeling more emotionally grounded.

R4: “I’ve started to dress a little bit more like I do at home outside, so I somehow make myself visible to others. I think I’ve tried to hide, or I think, I know, that in the past I’ve hidden from my workplace and friends and family. I have a self-perception of who I am, and I have always thought that everyone else can see that just as well as I can see it. I also thought that no one liked me for that. So, I hid in some ways, maybe dressed differently or lied about who I am to others. It’s been tough to have to do that, but now it’s completely different. I dress how I want to and I’m not afraid to express myself anymore.”

Respondents also reflected on how treatment had helped them do things that they previously had a hard time doing because of overwhelming emotions that previously were hard to manage, thereby improving daily functioning.

R4: “I used to avoid certain places or situations to avoid stress or experiencing difficult feelings. Now it’s not on the same level, but I still have it somehow. I remember places where I really feel very uncomfortable. It’s kind of in the back of my mind, but I can still manage to get through without much stress.”

Even though the emotion focus was experienced as beneficial by most respondents, many also described feeling like it was hard to put into practice. When they needed adaptive emotion regulation strategies the most, they were hardest to use which underscores the need for continued support in developing and implementing adaptive emotion regulation strategies.

R1: “To have some kind of rules is the wrong word, but a model, to process intense emotions can be useful. The difficult thing about it is that you have the model without knowing when it is good to implement, and when it is good to implement then it is difficult to think about it.

Respondents’ recounts showed how increased emotional understanding and clarity impacted their daily life, supporting them in navigating social interactions and affirming their identities externally. However, this process of gaining an increased understanding had limitations as these insights were difficult to translate into everyday situations.

### 3.3 Theme 3: possibilities for and limitations in social support

This theme encompasses respondents’ reflections on the impact of inviting a close other to the parallel support intervention and the perceived impact this had. The optional component of inviting a close other revealed complex dynamics around help-seeking, social support, and vulnerability.

For those who opted to invite a close other to parallel treatment, experiences varied where one respondent did not feel like their close other’s participation had affected their interaction or relationship. More commonly though, respondents described positive experiences of having a close other involved in treatment.

R2: “It was really nice to be able to talk about the fact that we both had modules and the stuff that related, so it was nice to have someone doing that and it became a bit more of a conversation about it.”

Beyond facilitating conversations, respondents reflected on how a close other’s participation was perceived as helpful for the close other themselves, and that the content of the support modules had helped their close other to understand themselves and the respondent better.

R5: “I invited my mom, and she has always been very supportive of me, but I think she was very happy with it anyway, that she was able to get a better understanding of her emotions and understand things better I think.”

In this way, the option to invite a close other was generally perceived as positive by those who opted to do so, both as a close other’s participation affected interaction between the respondents and their close other, but also as this affected the close other directly and their self-understanding.

While some respondents invited close others and found the joint engagement enriching, others described emotional barriers or structural challenges that prevented them from doing so. In some instances, this was related to experiencing close others as critical and unsupportive, R1: “I guess the biggest reason is that my family is not supportive of this.”. However, others described more ambivalent feelings and difficulties asking for help affecting their decision.

R3: “Okay, now I’m taking someone’s time. Do they want to spend this time? So, it’s not necessarily that I don’t want to, but at the same time it’s that I don’t want to. I avoid asking someone, for example, ‘Okay, do you want to do this and help?’”

Respondents’ descriptions revealed an interpersonal dimension of therapeutic change, highlighting both the relational potential of parallel interventions and the barriers TGD individuals may face when accessing support, both with close others being openly critical thus reducing their supportive potential, but also internal struggles affecting the extent to which support felt obtainable. This suggests that while social support can be a powerful therapeutic resource, its availability is shaped by context, past experiences, and experienced emotional safety, which should be considered in treatment provision.

### 3.4 Theme 4: barriers and bridges to treatment engagement

This theme encompasses respondents’ recounts on aspects permeating the treatment that impacted respondents’ ability to engage in treatment. While respondents found many aspects of the treatment valuable, they also reflected on limitations in treatment structure and delivery.

Several respondents emphasized that having content explicitly written with TGD individuals in mind contributed to a sense of belonging and visibility, fostering engagement and making the intervention feel relevant to their lived experiences.

R2: “It was very rewarding that there were many concrete examples that felt very correct and relevant for trans people. It felt very based in something real, that it was not taken out of thin air, but things I recognize, things I have talked to other trans people about. I felt more seen in knowing that there is understanding in psychiatry for these things, it felt very hopeful and positive.”

However, one respondent described feeling the opposite, and perceived examples as oblivious. For this respondent, this was described as a barrier impeding them from wanting to fully engage in treatment.

R1: “I found the material I had to work with very optimistic in relation to what it’s like to be a trans person in today’s political and social climate”.

A majority of the respondents expressed a desire for more face-to-face interaction or real-time discussion with their treatment provider, feeling that written feedback alone was insufficient for deeper engagement.

R2: “There was a lot that felt very valuable. A lot to go through, a lot to process from module to module. It would have been nice to just sit down and talk through it.”

Others described challenges related to the content’s intensity or focus, which sometimes felt misaligned with their personal experiences or needs which reduced the perceived relevance of treatment content.

R5: “I think, there weren’t enough examples in my everyday life for me to really go in depth with it, that my situation is more low-key stress. It felt like this was about someone who had it very chaotic or was kind of in the middle of a lot and it’s not really like that for me.”

This highlights the importance of flexibility and personalization in digital interventions. Although the treatment aimed to be broadly relevant to TGD individuals, variation in mental health needs, life circumstances as well as relational dynamics shaped how well respondents could connect with the material, which respondent 4 also reflected on.

R4: “I think there was a lot of focus on relationships in this study, there was a lot involving others. I was okay with that, but I personally felt that maybe that wasn’t what I needed to focus on.”

Another aspect respondents commonly reflected on was the timing of treatment. While some felt like treatment was administered at an optimal point in time, many respondents wished that treatment would have been provided either earlier or later in their own processes of identity development and transition. This was often related to feelings of being too early or too far along in their own social transition, suggesting that treatment content was perceived to be most helpful during an active process of social transitioning, R5: “Treatment would perhaps have been most helpful if it had come just when I came out.”

When respondents felt like treatment was well-timed, they often related this to their ongoing assessment of gender dysphoria. Treatment was then described to alleviate strain during long wait times between meetings or to help facilitate conversations within sessions in the assessment.

R10: “It had a kind of purpose to survive the time to the assessment in some way, to do something, to talk, to be able to discuss, to understand certain things for myself.”

These recounts highlight several possible adaptions to treatment that might enhance the experienced usefulness of treatment including more direct contact with a treatment provider, personalization of treatment in relation to life circumstances and timing in relation to social and medical transition.

## 4 Discussion

This study explored the needs and experiences of TGD individuals participating in an internet-delivered emotion regulation intervention specifically developed for TGD people. Using reflexive thematic analysis, four themes were constructed that provide insight into motivations for treatment, experiences of treatment, and perceived outcomes. The findings contribute to a growing understanding of how digital mental health interventions can support TGD individuals when tailored to their lived realities.

Participants often entered treatment seeking greater emotional clarity, and many found that the intervention supported self-exploration and identity development in ways that were personally meaningful. As emotional awareness increased, participants reported positive behavioral and relational changes, including improved communication and more authentic gender expression. The optional component of treatment of inviting a close other was related to both benefits and barriers, underscoring the ambivalent role of social support in the lives of many TGD individuals. Although treatment content was generally perceived as meaningful and helpful, participants also encountered challenges related to the intervention’s structure and delivery, particularly when content felt misaligned with their personal context or because of a lack of interpersonal contact. Collectively, these themes highlight the therapeutic potential of identity-affirming digital interventions, while also pointing to the importance of flexible delivery, relational sensitivity, and the centrality of identity in the emotional lives of TGD people.

The fact that participants often entered treatment with diffuse or general emotional needs, seeking greater clarity, emotional insight, or tools to navigate overwhelming feelings align with prior research showing that emotional regulation difficulties are common among TGD individuals and are often compounded by experiences of stigma, discrimination, and dysphoria (33, 34). Importantly, emotional needs were not necessarily framed as experiencing psychiatric symptoms or problems, but rather as part of a broader effort to make sense of the self. This further underscore the importance of how an intervention is framed and presented to potential participants. As participants did not frame their needs in clinical terms, they may not have sought out an intervention described strictly in terms of treatment or therapy. In relation to more diffuse and general needs, a framing that emphasizes support for living as a TGD individual, developing resilience, or building emotional skills might be perceived as more accessible (35), terms like support program, psychoeducation, or skills training may resonate more with the community and reduce potential misconceptions associated with seeking psychological care. Ultimately, this framing can help position the intervention as a resource for empowerment and growth, rather than solely a response to distress.

Results, particularly those described in Theme 2, From Emotional Insight to Change, provide evidence that increased emotional understanding was not confined to internal experiences as participants also described tangible shifts in how they related to others, navigated social spaces, and expressed their gender identity in working with content that aimed to advance their own emotional understanding. These findings support the conceptualization of emotion regulation not only as a set of skills, but as a catalyst for broader personal growth and behavioral change (36). In the context of TGD identity, the ability to regulate emotions may in this way help counteract internalized stigma and facilitate more authentic self-expression (13).

The results emphasize the complex social contexts in which TGD individuals engage in treatment. While the inclusion of a close other was beneficial for some, others described ambivalence, discomfort, or lack of access to affirming relationships. This underscores the relational strain many TGD individuals face and reflects broader findings on the role of minority stress and family rejection in shaping mental health, which aligns with previous research (37). Importantly, this theme suggests that treatment programs should offer flexible, non-prescriptive approaches to including important others. When included, most participants expressed that this was a positive addition to their treatment, but in designing interventions in this way, one may need to consider the emotional strain and risks associated with disclosing one’s identity or needs to others and actively support TGD individuals in doing so.

Participants generally felt that examples given in the treatment modules felt applicable to their experiences and made them feel seen, which affected treatment engagement positively for most participants. However, one participant had the opposite reaction to examples given in the modules, and felt that these were simplified, overly positive and therefore came off as oblivious. In this experience, this participant described a reduced interest in engaging fully in treatment. This highlights the importance of representation and cultural relevance in therapeutic content, as our results support that this affects engagement positively when perceived to be present and negatively when lacking. Participants who perceived the intervention as a rare space within healthcare services in which their identities were reflected and affirmed described that this enabled reflection on gender identity, self-worth, and belonging. These finding echoes research on culturally adapted interventions, which demonstrates that representation and affirming language significantly enhance psychological safety and perceived helpfulness for marginalized groups (38, 39). The treatment’s explicit centering of TGD experiences contributed to a sense of legitimacy and visibility for many respondents in contrast to the often pathologizing mainstream mental health services (40).

Nevertheless, the format of the intervention introduced certain limitations. While online formats increase accessibility, our findings point to the importance of flexibility and personalization in digital interventions. **Table 4** summarizes the experienced constraints of our mode of treatment delivery and potential adaptations to address them.

**Table 4:** Constraints in treatment delivery and potential adaptations.

Constraints of digital intervention	Potential adaption of treatment
Lack of real-time interaction made it difficult to process complex emotional content.	Blended delivery models that incorporate live support to enhance engagement and therapeutic depth (41, 42)
Content was presented in modules in a fixed order; participants at times experienced modules to be misaligned with their needs.	Providing treatment modules in a modular way, based on individual needs rather than in a fixed order. Some participants may initially require support with emotional regulation in the context of gender dysphoria, while others may benefit more from modules focused on interpersonal functioning, identity development, or self-acceptance, which are areas previous research support as being important to target (43, 44).
Participants had completed on average 7.2 modules out of the 10 available modules.	Identification and isolation of key components of the treatment that could be offered in shorter formats or adapted for different delivery contexts. For instance, a brief emotion regulation intervention only using contents from module 3–5 could be made available as a stand-alone intervention, or other selected modules could be integrated into broader support programs. This would increase the intervention’s flexibility and reach, allowing for targeted support depending on individual goals and time constraints.

Further, Theme one describes how participants began developing a language for understanding their emotional experiences during the intervention. This not only helped individuals gain insight into their own emotions, but also facilitated the identification of new psychological, social or relational needs that may not have been fully recognized before, which aligns with previous research on mechanisms of emotion regulation treatments (36). This further underscores the value of allowing participants to navigate the treatment in a non-linear way, adjusting the sequence of modules based on evolving insights and priorities, and routine outcome measures to enhance treatment effect (41, 45).

### 4.1 Implications for practice and research

This study offers several practice-oriented insights. First, interventions for TGD individuals should be culturally specific and provide representation, embedding identity-affirming language and examples throughout therapeutic content. Second, there is a need for flexibility in treatment delivery. The option for synchronous support or tailored treatment provision may improve engagement, especially for individuals with varying levels of emotional need. Third, efforts to involve close others should be grounded in sensitivity to family and social dynamics and should avoid assuming that affirming relationships are available or safe for all TGD individuals.

For researchers and intervention developers, these findings highlight the importance of co-production of treatments with TGD communities, ensuring that interventions not only address emotional regulation as a clinical goal, but also as a means of supporting gender identity, self-advocacy, and relational efficacy. A key implication of this study is the need to broaden the spectrum of available interventions for TGD individuals, moving beyond a predominant focus on medical interventions. While gender-affirming medical care is essential for many, there is also a need for interventions that address the psychological, emotional, and social dimensions of living as a TGD person, especially for those who may not require or have limited access to medical care (33, 44). Emotion regulation support, psychoeducation, and community-informed therapeutic interventions can provide critical tools for navigating the challenges of minority stress, identity formation, and social affirmation.

### 4.2 Strengths and limitations

This study’s findings should be considered in light of several limitations. The sample does not reflect the full diversity of TGD individuals, particularly across different cultural or socioeconomic backgrounds as the current sample predominantly grew up in Sweden and had fairly high levels of academic achievement. Also, all participants were patients at a specialized health care clinic. Beyond this, one should consider that participants were self-selected and had a higher completion rate of treatment compared to those who did not choose to participate in the interviews. Our data may therefore reflect more positive experiences than would be present in a broader sample. Additionally, the perspectives of close others were not included, limiting understanding of the parallel component’s full impact.

As a qualitative study, generalizability of results is not the main goal, but rather to offer rich, contextualized insights into the lived experiences of TGD individuals in a novel intervention format. A strength of the current study was its consideration of trustworthiness of the analysis, which was improved by the use of the 15-point checklist of criteria for good thematic analysis (30). The authors provided differing expertise from varied academic and clinical disciplines with extensive clinical experience with TGD individuals that aided understanding of participant’s recounts of treatment experiences. Future research might should use these findings in larger and more diverse samples and explore potential adaptions in treatment provision described above that further need to be evaluated in efficacy studies.

## 5 Conclusion

This study contributes to the growing field of identity-affirming mental health interventions by illuminating how TGD individuals experience and respond to an internet-delivered emotion regulation treatment. Participants described the intervention as valuable for developing emotional insight, supporting self-expression, and affirming identity, though some noted challenges related to structure and access to support. These findings suggest that effective interventions for TGD populations must be emotionally responsive, culturally grounded, and flexibly delivered, recognizing both the psychological and social dimensions of TGD individuals’ mental health. Continued research and community-informed innovation are needed to ensure that digital mental health tools can fully meet the needs of those they aim to serve.

## Data Availability

The datasets presented in this article are not readily available because The dataset consists of transcripts of interviews, which cannot be shared in full while maintaining respondent anonymity. Requests to access the datasets should be directed to markus.bystrom@ki.se.
