# Supplementary material for: “You get a better idea of what you want to do with your life”: needs and experiences of transgender and gender diverse individuals participating in an internet delivered emotion regulation treatment
Source: Front Psychiatry. 2026 Feb 5;17:1659076. doi: 10.3389/fpsyt.2026.1659076 (PMC12916390; doi:10.3389/fpsyt.2026.1659076)
Supplement: Supplementary file 1 [file DataSheet1.pdf]

Supplementary material 1. Interview guide

Introduction
The focus of this interview will be your experience of the treatment you recently participated in. This may include both how you were positively affected and what may have affected you negatively. We are interested in your experience, and there are no right or wrong answers.

Opening Questions
I will start with a few questions about the time before treatment began.

1. What led you to seek treatment?

- What needs did you have that you hoped the treatment could meet?

Experience of the Treatment
2. How did you experience the treatment?

- Was the content relevant in relation to your needs?

- If you discontinued treatment, what was the reason?

3. How did you experience working with minority stress?

- Do you feel this was relevant for you? In what way?

4. How did you experience working with emotion regulation?

- Do you feel this was relevant for you? In what way?

Effect of the Treatment
Now I would like to ask some questions about changes the treatment has brought.

6. What has changed after the treatment?

- What positive changes you have experienced?

- What negative changes have you experienced?

- How has the treatment affected how you experience your emotions?

- How has the treatment affected how you relate to or understand minority stress?

- What new skills have you developed?

- How did the treatment affect your well-being or your everyday life?

7. What have you found helpful?

- What was your most important take away from the treatment?

8. What needs do you have after the treatment?

- What did the treatment not help you with?

Treatment evaluation

Now, in the final part of the interview, I would like to ask some more concrete questions about how you experienced the content of the treatment.

9. How did you experience having / not having a support person in the parallel treatment?

- What was the reason you chose to invite a support person/ not invite a support person?

10. How did you experience the texts in the treatment?

- Were they helpful? Were they too long, too short or a good length?

11. How did you experience the exercises and worksheets in the treatment?

- Were they helpful? Were they too complicated or too simple?

12. How did you experience your contact with the therapist?

- Did you get the help you needed, and did the format work for you?

13. How much time would you estimate you spent working on the treatment each week?

- Do you think the time you spent was reasonable?

14. Do you think the treatment was practically feasible for you, given your life situation?

- If you could do the treatment again, would you want to do it at a different time? Earlier, later, unrelated to the assessment of gender dysphoria, etc.

15. Do you feel that the treatment influenced your assessment of gender dysphoria in any way?

- Was your approach during the assessment affected?

- Did you feel limited in the treatment or the assessment by doing them simultaneously?

- Do you feel you were able to use what you worked on in the treatment during sessions in the assessment?

Closing

You have shared your experience of the treatment you recently underwent. Our interview is almost over, but before we finish, I’d like to ask if there are any new thoughts or reflections that have come up during our conversation that you would like to share with me?

Thank you for sharing your experiences. We are grateful that you wanted to share all this with us and that you participated in the studies!
